# Associations between Urinary Phthalate Metabolites and Serum Anti-Müller Hormone Levels in U.S. Men Based on National Health and Nutrition Examination Survey 2003–2004

**DOI:** 10.3390/ijerph14121513

**Published:** 2017-12-05

**Authors:** Ningning Li, Yaqi Li, Hao Meng, Hanqing Sun, Di Wu

**Affiliations:** 1 State Key Laboratory of Reproductive Medicine, Institute of Toxicology, Nanjing Medical University, Nanjing 211166, China; lnning1235@163.com (N.L.); m18851729097_1@163.com (Y.L.); leonhardt_k@163.com (H.S.); 2 Key Laboratory of Modern Toxicology of Ministry of Education, School of Public Health, Nanjing Medical University, Nanjing 211166, China; 3 School of Geography and Oceanography Sciences, Nanjing University, Nanjing 210023, China; menghao09@163.com; 4 Key Laboratory of Reproductive Medicine, Institute of Toxicology, Nanjing Medical University, Nanjing 210029, China

**Keywords:** phthalate metabolites, anti-Müller hormone, NHANES, man

## Abstract

Anti-Müller hormone (AMH) plays an important role in reproductive development and has a wide potential clinical application value. Phthalates have been widely found in human living environment and have negative effects on human reproduction. This study aimed to explore the relationship between urinary phthalate metabolites and serum AMH level in the general male population. Cross-sectional analyses were performed with a population of 489 men aged more than 12 years who participated in National Health and Nutrition Examination Survey (NHANES) 2003–2004 by Centers for Disease Control and Prevention, the United States. NHANES public data (demographic and socioeconomic information, examinations, and laboratory tests) were analyzed using Kruskal-Wallis test, Wilcoxon test and multivariable regression. Results showed that the urine concentration of mono (3-carboxypropyl) phthalate (MCPP) of 12–20 age group was significantly positively correlated with serum AMH concentration in the model without any covariates (*p* < 0.05). In the 60-year-old group, the monomethyl phthalate (MEP), mono (2-ethyl-5-carboxypentyl) phthalate (MECPP) concentrations were significantly correlated with serum AMH concentrations in models both with and without covariates (all *p* < 0.05). It could be concluded that exposure to phthalates might have negative effects on AMH level, especially in seniors. AMH could be used as a marker of exposure to phthalates in aged males. How exposure to phthalates affected AMH level and what the potential long-term health consequences of their relationship are needs more investigation.

## 1. Introduction

Phthalates are environmental endocrine disruptors widely used in human lives [1,2,3]. They have been classified as endocrine disrupting chemicals (EDCs), the exposures of which had been discovered to lead to disorders in humans—inducing childhood obesity, respiratory diseases, neuropsychological disorders and fertility problems [4]. One of the most common phthalates was di-2-ethylhexyl phthalate (DEHP) [5]. 

Animal studies have revealed potential reproductive and developmental toxicities of phthalates. It had been found that di-n-butyl phthalate (DBP), DEHP and butyl benzyl phthalates (BBzP) intrauterine exposure caused anogenital distance reduction in offspring male rat [6]. Prenatal exposure to DEHP led to decreased androgen receptor expression and Anti-Müller hormone (AMH) levels in testes in offspring male mice [7]. Human studies also proved the reproductive and developmental toxicities of phthalates. In men, phthalates exposure had been associated with testicular dysgenesis syndrome. Phthalates exposure also could reduce sperm quality and testosterone level, indicating that they might play a role in male infertility [4]. The associations between phthalate exposure and adverse male genital development in human male newborns had been reported [8]. A reduction of the anogenital index had been observed in infant boys with increasing metabolites monomethyl phthalate (MEP), mono-butyl phthalate (MBP), monobenzyl- and mono-isobutyl phthalate levels in maternal urine during late-pregnancy [9]. Mono (2-ethylhexyl) phthalate (MEHP) levels in maternal urine were associated with a decrease in testosterone/estrodial and progesterone and inhibited B levels in fetal serum [10]. MEHP and the hydrolytic metabolite of DEHP measured in urine of 221 males were negatively correlated with testosterone, estradiol, and the free androgen index [11].

AMH is a glycoprotein secreted by the testes immature support cells and ovarian granule cells. Its known physiological role is regulating the gender differentiation, the development of male and female reproductive systems, and the maintenance of normal functions [12,13]. AMH is a potential clinical marker, especially in the field of reproduction. Semen AMH help indicate infertility in males. Prospective cohort studies have shown that semen AMH levels were positively correlated with sperm count [14] and acrosin [15]. Serum AMH has been used to study testicular function during the prepubertal period in males [16,17]. AMH could indicate gonadotropin actions in the testis of children and adolescents with disorders of the gonadal axis [18]. 

Only a few reports have studied the relationship between phthalate exposure and AMH level. Generally, phthalates exposure has been found to reduce the secretion of AMH in rodents. MEHP could reduce the level of AMH in the rat fetal testis [19]. DEHP could negatively influence the testis AMH level in male mice pups [7]. In order to evaluate the association between human phthalates exposure and AMH level directly, we chose to focus on male urine phthalate metabolite levels and further to analyze their associations with the serum AMH level. 

## 2. Subjects and Methods

### 2.1. Study Population

The present data included measurements from two years of NHANES (National Health and Nutrition Examination Survey), 2003–2004. NHANES was a representative survey research program to assess the health and nutritional status of adults and children in the United States of America [20]. The data were collected by means of demographic, interview, examination, questionnaire, and laboratory tests. NHANES received approval from the National Center for Health Statistics Ethics Review Board, and informed consent was obtained for all participants (Protocol #98-12). Our analyses were based on the NHANES public data. We excluded participants whose covariates were missing. A total of 489 male participants were included in final regression analyses.

### 2.2. Urinary Phthalate Metabolites and Serum AMH Levels

Detailed urine and serum sample collection and processing had been instructed in the NHANES Laboratory/Medical Technologists Procedures Manual (LPM). We selected 13 kinds of phthalate metabolites which are critical metabolites in human urine as presented by the American Centers for Disease Control and Prevention [21]. In Division of Laboratory Sciences, National Center for Environmental Health, Centers for Disease Control and Prevention, high performance liquid chromatography-electrospray ionization-tandem mass spectrometry (HPLC-ESI-MS/MS) (Waltham, MA, USA) had been utilized for the quantitative detection in urine of the following phthalate metabolites: MBP, monocyclohexyl phthalate (MCNP), MEP, MEHP, monoisononyl phthalate (MiNP), monooctyl phthalate (MCOP), monobenzyl phthalate (MBzP), monomethyl phthalate (MnMp), MCPP, mono (2-ethyl-5-hydroxyhexyl) phthalate (MEHHP), mono (2-ethyl-5-oxohexyl) phthalate (MEOHP), mono-isobutyl phthalate (MiBP) and MECPP. A summed measure of DEHP metabolites (ΣDEHP)—including MEHP, MEHHP, MEOHP, and MECPP—was also measured. The sum was created based on nanomolar concentrations of each metabolite using the following formula: ΣDEHP = (MEHP/278) + (MEHHP/294) + (MEOHP/292) + (MECPP/308). Final ΣDEHP concentrations were in micromoles per liter. Values below the limit of detection (LOD) were replaced with a value of the LOD divided by the square root of 2. Urinary phthalate metabolite levels were corrected by dividing urinary phthalate concentration with creatinine *0.01 (μg/g creatinine). 

The Beckman Coulter Gen II AMH ELISA was used to measure AMH levels in the serum samples from male participants by the National Center for Environmental Health, Centers for Disease Control and Prevention [22]. 

### 2.3. Covariates

We examined the following as potential confounding variables: age, race/ethnicity, education level, family income to poverty ratio (PIR, showing a ratio of family income to poverty threshold), and six-month sampling period (a proxy variable for season) from the in-home demographic questionnaire. Body mass index (BMI) was from examination data. There were four age categories: 12 to <20; 20 to <40; 40 to <60; ≥60. Four race categories were: Non-Hispanic White; Non-Hispanic Black; Mexican American; Other Race; Other Hispanic. There were three education level categories: Less than high school; High school/GED (General Equivalency Diploma); More than high school. Four BMI categories were: Underweight (<18.5); Normal weight (18.5–24.9); Overweight (25–29.9); Obesity (>30). There were two categories in PIR: PIR category < 1; PIR category ≥ 1. Two sampling period categories were: Winter months (1 November–30 April); Summer months (1 May—31 October).

### 2.4. Statistical Analysis

Both unweighted and weighted analyses were used in our analysis. The associations between urinary phthalate metabolites and serum AMH were examined using multivariable regression.

First, we examined differences in serum AMH levels by category of population characteristics using Kruskal-Wallis test and Wilcoxon test. The Kruskal-Wallis test was used to compare the serum AMH levels of subgroups of age, race, education level, BMI, and then multiple comparisons among groups were performed. Wilcoxon test was used to compare the serum AMH levels of subgroups of PIR category and sampling season. The multivariable linear regression was used to estimate associations between urinary phthalate metabolites and serum AMH. All urinary phthalate metabolites and serum AMH data were transformed into their natural logarithm (ln) because of the right-skewed individual distributions. Crude regression models were created, adjusted by urinary creatinine only, and a full model was additionally adjusted by race/ethnicity (categorical), body mass index (BMI), and education level (categorical). In each model, urinary phthalate metabolites and serum AMH level were treated as continuous variables. Statistical analysis was performed with STATA Version 13.0 (Stata Corp., College Station, TX, USA). A probability value of less than 0.05 was considered statistically significant.

The selected 13 kinds of phthalate metabolites actually were related to each other. The correlations among 13 kinds of phthalate metabolites are shown in Table 1. The correlation coefficients among MCOP, Mono, and MiNP; and the correlation coefficients among MEHHP, MEOHP, MECPP, and MEHP were greater than 0.8. In order to reduce the collinearity of the model and improve the fit of the model, after principal components analysis, we chose MiNP and MECPP as representatives and excluded Mono, MEHP, MCOP, MEHHP, and MEOHP. So the eight phthalate metabolites we further analyzed included MBP, MEP, MBzP, MnMp, MCPP, MiBP, MiNP, and MECPP. The heterogeneity among different stratifications was not found.

## 3. Results

Population characteristics of 489 participants are presented in Table 2. In weighted analysis the age of the participants was mainly 40–60 years old. In unweighted analysis, the age of participants mainly lied between 12–40 years. In weighted analysis, 73.7% of participants were Non-Hispanic White, 10.63% were Non-Hispanic Black, 9.24% were Mexican American, and 3.3% were Other Race, 3.13% were Other Hispanic. Nearly half of the participants’ education level was above high school. 31.05% participants were at normal weight, 33.21% were overweight, 32.57% reached obesity. 83.51% was in the category with family PIR ≥1. In unweighted analysis, 44.38% of participants were Non-Hispanic White, 24.34% were Non-Hispanic Black, 25.97% were Mexican American, and 2.04% were Other Race, 3.27% were Other Hispanic. 48.88% had less than high school education, 31.7% had more than high school. 35.58% participants were at normal weight, 31.08% were overweight, and 25.77% reached obesity. 75.26% were in the category with family PIR ≥1.

Besides population characteristics, the AMH concentration means of different subgroups were also shown in Table 2. In unweighted analysis, the main effect of age was significant on AMH level (*p* < 0.001). Specifically, the group with age more than 60 had significant lower AMH level comparing to 12 to 20 group, 20 to 40 group, and 40 to 60 group (all *p* < 0.05), and the 40 to 60 group had lower AMH levels compared to 12 to 20 group (*p* < 0.05). Similarly, the main effect of race was significant on AMH level (*p* < 0.001). Specifically, the Non-Hispanic Black group had significantly higher AMH level compared to Non-Hispanic White group, Mexican American group, and Other Race group (all *p* < 0.001). The differences of serum AMH levels among BMI subgroups were statistically significant (*p* < 0.001). Specifically, the normal weight group had significantly higher AMH levels compared to overweight group and obese groups (both *p* < 0.001), the underweight group also had significantly higher AMH levels compared to overweight group and obese group (both *p* < 0.001). The main effect of education level on the serum AMH level was also significant (*p* < 0.05), “more than high school” group had significantly lower AMH level compared to the “less than high school” group and “high school/GED” group (both *p* < 0.05).

Geometric means and selected percentiles of creatinine-corrected urinary phthalate metabolite levels in weighted analysis were presented in Table 3. The unweighted analysis results were shown in Table 4. In both models, MEP level was highest among the urinary phthalate metabolites while ΣDEHP was the lowest.

The crude associations between in-transformed urinary phthalate metabolites and AMH concentrations are presented in Table 5. In males aged 12–20, only MCPP had a significantly positive association with AMH (β = 0.37, 95%CI = 0.05–0.69, *p* = 0.023). When age was above 60 years, there was a significantly positive association between MEP and AMH and an inverse association between MECPP and AMH (β = 0.13, 95%CI = 0.03–0.23, *p* = 0.014; β = −0.21, 95%CI = −0.38–0.04, *p* = 0.017 respectively). After the associations had been adjusted by race/ethnicity, education level, and BMI (Table 6), the association between MCPP and AMH was not significant any more in age 12–20. However, in those aged over 60, the associations between MEP and AMH and MECPP and AMH were still significant (β = 0.11, 95%CI = 0.01–0.21, *p* = 0.029; β = −0.20, 95%CI = −0.3–0.03, *p* = 0.020 respectively). No significant relationships had been observed in either age 20–40 or age 40–60.

## 4. Discussion

In this study, we analyzed the relationships between serum AMH concentration and the urine phthalate metabolite levels in U.S. men based on the public data from National Health and nutrition examination survey 2003–2004. 

The regression was not significant between phthalate metabolite concentrations and AMH in the WHOLE population. The main effect of age on serum and semen AMH level has been documented [23] and our results show that the level of AMH at the age above 60 is the lowest, as reported in the cross-sectional study [24]. As a result, we further divided the population into different age groups and did find significant regression in some age groups. 

For example, when multiple regression analysis was used with age as an independent variable, the association between MCPP and AMH was not significant. However, there was a significantly positive association between MCPP and AMH in 12–20 age group, but not in 20–40, 40–60, and senior age groups. The significant regression in specific age group has been attenuated when the population is considered as a whole. Heterogeneity analysis confirmed there was no interaction of age and phthalate metabolite concentrations. 

The urine MCPP level of 12–20 age group was positively correlated with serum AMH concentration in the model without any covariates, but the correlation was not statistically significant after covariates of race/ethnicity, body mass index (BMI), and education level were considered. In the 60-year-olds, the MEP concentration was positively correlated with serum AMH level in models with or without covariates. MECPP concentration was negatively correlated with AMH level. There were no significant correlations found in both models in the other two age groups.

There were two animal studies that explored to the effects of phthalate exposure on AMH. Chauvigné [19] showed that MEHP exposure reduced the number of germ cells and increased germ cell apoptosis; although it had no effect on the number of stromal cells and supporting cells, DEMP exposure impaired their function and reduced the secretion of AMH and testosterone. The study conducted by Xi and his colleagues [7] found that DEHP exposure reduced the weight of the testis, the sperm count, and the secretion of FSH and testosterone and AMH gene expression. The two studies together show that DEHP downgrades AMH levels.

In our study, MECPP concentration was negatively correlated with AMH concentration in males over 60 years of age. Since MECPP was one of the metabolites of DEHP, which was indirectly consistent with the results of two animal studies. 

In addition, a positive correlation between urinary MEP concentration and serum AMH concentration was found in the regression model of men over the age of 60 years, which has not been mentioned in other studies. One reported study of 168 adult males showed that the concentration of MEP in urine was positively correlated with sperm DNA damage [25]. Jonson and his colleagues analyzed 234 young men samples, found that urine MEP was associated with the reduction of active sperm count and serum luteinizing hormone level [26]. Duty’s work [27] showed a significant positive correlation between urinary MEP level and serum testosterone concentration. As a result, MEP has been closely related to AMH, but its specific role which led to the correlation with AMH observed in this study is still unknown, and more in-depth studies are needed to clarify this correlation.

Although not all metabolites selected had been associated with AMH level, and some correlations were only found in age group over 60 years of age, on the whole, phthalate exposure had a certain impact on male AMH secretion. Phthalates had been known to have negative effects on the testis, including inhibiting the development of sperm, altering reproductive hormone levels, and even disturbing the functions of active substances in the body such as thyroxine and vitamin D [28,29,30,31,32]. This accumulating evidence could partly explain relationship between phthalates and AMH, but the mechanism underlying had not been very clear yet. Phthalates might alter AMH level though affecting active substances in testis and other reproductive tissues. Or it might alter the level of AMH directly and thus damage the normal functions of the testis. There might be other cytokines involved, which also need further studies to prove. Furthermore, the relationship was found only in the age group over 60 years of age. Whether it is due to the result of cumulative effects or physiological changes also needs to be further explored.

## 5. Conclusions

In this study, we investigated the relationships between urine phthalate metabolites and AMH levels and found significant associations between AMH and urinary MEP and MECCP, specifically in the aged group. AMH could be used as a marker of phthalates exposure in aged males. How phthalates exposure affected AMH levels and what the potential long-term health consequences of their relationship are needs more investigation.

## Figures and Tables

**Table 1:** Correlation matrix among phthalate metabolites.

Urinary Analyte	MBP	Mono	MEP	MEHP	MiNP	MCOP	MBzP	MnMp	MCPP	MEHHP	MEOHP	MiBP	MECPP
MBP	1												
Mono	−0.01	1											
MEP	0.13	0.03	1										
MEHP	0.10	0.26	0.13	1									
MiNP	0.01	0.92	0.02	0.33	1								
MCOP	−0.01	0.96	0.02	0.27	0.95	1							
MBzP	0.51	−0.07	0.13	0.08	−0.06	−0.08	1						
MnMp	0.15	0.34	0.11	0.41	0.37	0.34	0.14	1					
MCPP	0.32	0.21	0.07	0.25	0.27	0.22	0.19	0.28	1				
MEHHP	0.35	−0.00	0.03	0.33	0.05	−0.00	0.27	0.29	0.27	1			
MEOHP	0.30	0.01	0.01	0.36	0.13	0.08	0.21	0.33	0.39	0.86	1		
MiBP	0.39	0.13	0.09	0.20	0.14	0.13	0.21	0.15	0.28	0.22	0.32	1	
MECPP	0.23	0.06	0.01	0.91	0.11	0.07	0.16	0.29	0.29	0.93	0.83	0.17	1

Note: MBP: mono-butyl phthalate; MEP: monoethyl phthalate; MEHP: Mono (2-ethylhexyl) phthalate; MiNP: monoisononyl phthalate; MCOP: monooctyl phthalate; MBzP: monobenzyl phthalate; MnMp: monomethyl phthalate; MCPP: mono (3-carboxypropyl) phthalate; MEHHP: mono(2-ethyl-5-hydroxyhexyl) phthalate; MEOHP: mono(2-ethyl-5-oxohexyl) phthalate; MiBP: mono-isobutyl phthalate; MECPP: mono (2-ethyl-5-carboxypentyl) phthalate.

**Table 2:** Distribution of demographic and laboratory characteristics in NHANES (2003–2004) males and their Anti-Müller Hormone means (*n* = 489).

Population Characteristics	*n*	Unweighted (%)	Weighted (%)	AMH, Unweighted Geometric Mean, (ng/mL)	*p*-Value
Age					<0.001
12 to <20	162	33.13	12.98	6.43	
20 to <40	116	23.72	34.10	4.94	
40 to <60	98	20.04	34.22	4.37 ^a^	
≥60	113	23.11	18.70	4.21 ^b^	
Race					<0.001
Non-Hispanic White	217	44.38	73.7	4.07	
Non-Hispanic Black	119	24.34	10.63	7.00 ^c^	
Mexican American	127	25.97	9.24	5.58	
Other Race	10	2.04	3.30	3.10	
Other Hispanic	16	3.27	3.13	5.72	
Education level					<0.05
Less than high school	239	48.88	26.79	5.78	
High school/GED	95	19.43	24.87	5.26	
More than high school	155	31.70	48.34	4.05 ^d^	
BMI, kg/m^2^					<0.001
Underweight (<18.5)	37	7.57	4.25	11.22 ^e^	
Normal Weight (18.5–24.9)	174	35.58	31.05	6.25 ^f^	
Overweight (25–29.9)	152	31.08	32.21	4.08	
Obesity (≥30)	126	25.77	32.57	3.91	
PIR category					0.68
<1	121	24.74	16.49	5.54	
≥1	368	75.26	83.51	4.92	
Sampling season					0.43
Winter months	241	49.28	43.31	4.71	
Summer months	248	50.72	56.69	5.45	

Note: ^a^ Significantly lower compared to 12 to 20 group (*p* < 0.05); ^b^ Significantly lower compared to the other groups (all *p* < 0.05); ^c^ Significantly higher compared to Non-Hispanic white group, Mexican American group and Other Race (all *p* < 0.001); ^d^ Significantly lower compared to Less than high school group and High school/GED group (*p* < 0.05); ^e^ Significantly higher compared to overweight group and obesity groups (both *p* < 0.001); ^f^ Significantly higher compared to overweight group and obesity groups (both *p* < 0.001). GED: General Equivalency Diploma; BMI: body mass index; PIR: family income to poverty ratio. AMH: Anti-Müller hormone. NHANES: National Health and Nutrition Examination Survey.

**Table 3:** Weighted, creatinine-corrected urinary phthalate metabolite concentrations (μg/g Creatinine) in males, NHANES 2003–2004.

Urinary Analyte	Geometric Mean	Selected Percentiles					Maximum
		25th	50th	75th	90th	95th	
MBP	13.61	8.82	13.03	21.17	31.28	44.20	3596.00
MCNP	0.47	0.32	0.45	0.62	1.00	1.41	3.73
MEP	107.32	37.90	97.19	265.55	595.04	1141.80	11,608.52
MEHP	2.09	0.92	1.67	4.12	8.68	20.86	277.38
MiNP	0.94	0.64	0.89	1.22	2.03	3.18	9.48
MCOP	0.95	0.65	0.90	1.23	1.93	2.88	7.62
MBzP	7.55	4.11	7.58	14.01	23.80	27.60	131.58
MnMp	1.74	0.80	1.35	3.18	7.54	11.19	692.60
MCPP	2.37	1.46	2.22	3.64	7.06	10.67	273.21
MEHHP	18.71	8.87	16.26	32.69	93.45	192.52	1186.05
MEOHP	12.45	5.71	10.89	24.30	67.33	122.35	1028.16
MiBP	3.01	1.76	3.06	5.29	8.75	13.47	159.50
MECPP	28.71	13.01	23.00	46.08	123.93	266.75	1303.78
ΣDEHP	0.23	0.11	0.18	0.35	1.05	1.98	10.99

ΣDEHP: A summed measure of DEHP metabolites.

**Table 4:** Unweighted, creatinine-corrected urinary phthalate metabolites (μg/g Creatinine) in males, NHANES 2003–2004.

Urinary Analyte	Geometric Mean	Selected Percentiles					Maximum
		25th	50th	75th	90th	95th	
MBP	14.71	9.27	14.76	23.34	34.33	47.65	3596
MCNP	0.44	0.31	0.43	0.6	0.95	1.24	3.73
MEP	110.7	40.49	97.73	253.97	643.59	1181.76	11,608.52
MEHP	1.97	0.83	1.52	4.06	9.56	20	277.38
MiNP	0.89	0.61	0.84	1.18	1.85	2.53	9.48
MCOP	0.9	0.62	0.86	1.19	1.83	2.49	7.62
MBzP	8.25	4.56	9.05	15.67	24.87	36.5	131.58
MnMp	1.65	0.75	1.28	3.06	7.78	12.5	692.6
MCPP	2.36	1.41	2.22	3.75	7.05	9.8	273.21
MEHHP	17.43	8.53	15.36	31.25	81.15	146.42	1186.05
MEOHP	11.7	5.67	10.83	21.09	52.63	95.37	1028.16
MiBP	3.16	1.9	3.24	5.5	9.05	14.55	159.5
MECPP	27.25	13.03	22.43	44.96	106.26	238.43	1303.78
ΣDEHP	0.21	0.11	0.18	0.35	0.89	1.59	10.99

**Table 5:** Associations between urinary phthalate metabolites and serum anti-Müller hormone level in males, NHANES 2003–2004 (unadjusted).

UrinaryAnalyte	Age 12–19 (*n* = 162)			Age 20–39 (*n* = 117)			Age 40–59 (*n* = 98)			Age ≥60 (*n* = 113)		
	β	(95%CI)	*p*	β	(95%CI)	*p*	β	(95%CI)	*p*	β	(95%CI)	*p*
MBP	0.20	(−0.22, 0.61)	0.347	0.00	(−0.43, 0.43)	0.972	−0.17	(−0.69, 0.35)	0.517	0.04	(−0.15, 0.22)	0.683
MEP	−0.14	(−0.33, 0.05)	0.141	0.04	(−0.21, 0.27)	0.761	−0.05	(−0.31, 0.21)	0.699	0.13	(0.03, 0.23)	0.014
MBzP	0.22	(−0.08, 0.52)	0.149	0.21	(−0.15, 0.58)	0.249	−0.03	(−0.43, 0.36)	0.867	−0.09	(−0.27, 0.10)	0.345
MnMp	0.07	(−0.17, 0.29)	0.559	−0.23	(−0.59, 0.12)	0.199	0.01	(−0.32, 0.35)	0.932	0.00	(−0.13, 0.13)	0.986
MCPP	0.37	(0.05, 0.69)	0.023	−0.22	(−0.70, 0.28)	0.379	0.25	(−0.14, 0.65)	0.204	0.00	(−0.16, 0.16)	0.986
MiBP	−0.06	(−0.35, 0.23)	0.681	−0.13	(−0.49, 0.24)	0.471	−0.01	(−0.38, 0.37)	0.966	−0.06	(−0.24, 0.13)	0.542
MiNP	0.17	(−0.27, 0.60)	0.446	0.33	(−0.23, 0.87)	0.237	−0.40	(−1.03, 0.24)	0.218	−0.03	(−0.34, 0.27)	0.820
MECPP	−0.09	(−0.35, 0.17)	0.483	0.19	(−0.10, 0.48)	0.199	0.01	(−0.26, 0.27)	0.967	−0.21	(−0.38, −0.04)	0.017

Note: The model had not been unadjusted by any covariant.

**Table 6:** Associations between urinary phthalate metabolites and serum anti-Müller hormone level in males, NHANES 2003–2004 (adjusted).

Urinary Analyte	Age 12–19 (*n* = 162)			Age 20–39 (*n* = 117)			Age 40–59 (*n* = 98)			Age ≥60 (*n* = 113)		
	β	(95%CI)	*p*	β	(95%CI)	*p*	β	(95%CI)	*p*	β	(95%CI)	*p*
MBP	0.20	(−0.19, 0.59)	0.316	0.02	(−0.49, 0.46)	0.948	−0.26	(−0.80, 0.27)	0.327	−0.03	(−0.21, 0.15)	0.725
MEP	−0.17	(−0.35, 0.00)	0.053	0.01	(−0.26, 0.25)	0.965	−0.23	(−0.29, 0.24)	0.833	0.11	(0.01, 0.21)	0.029
MBzP	0.21	(−0.08, 0.50)	0.15	0.28	(−0.11, 0.67)	0.162	0.10	(−0.33, 0.52)	0.653	−0.06	(−0.23, 0.12)	0.53
MnMp	0.06	(−0.15, 0.27)	0.562	−0.14	(−0.54, 0.25)	0.467	−0.02	(−0.38, 0.35)	0.933	0.00	(−0.13, 0.12)	0.941
MCPP	0.31	(−0.00, 0.62)	0.054	−0.17	(−0.68, 0.33)	0.491	0.33	(−0.09, 0.74)	0.126	0.02	(−0.13, 0.18)	0.788
MiBP	−0.03	(−0.31, 0.25)	0.828	−0.20	(−0.59, 0.19)	0.311	−0.07	(−0.45, 0.32)	0.735	−0.03	(−0.21, 0.15)	0.763
MiNP	0.19	(−0.26, 0.63)	0.405	0.37	(−0.20, 0.93)	0.201	−0.45	(−1.14, 0.25)	0.204	−0.07	(−0.37, 0.24)	0.669
MECPP	−0.04	(−0.29, 0.22)	0.781	0.15	(−0.15, 0.46)	0.322	0.03	(−0.26, 0.32)	0.847	−0.20	(−0.36, −0.03)	0.020

Note: The model had been adjusted for race/ethnicity (categorical), body mass index (continuous), and education level (categorical).
